# Adenoviral Transduction of Mesenchymal Stem Cells: In Vitro Responses and In Vivo Immune Responses after Cell Transplantation

**DOI:** 10.1371/journal.pone.0042662

**Published:** 2012-08-06

**Authors:** Oliver Treacy, Aideen E. Ryan, Teresa Heinzl, Lisa O'Flynn, Marese Cregg, Mieszko Wilk, Francesca Odoardi, Paul Lohan, Timothy O'Brien, Mikhail Nosov, Thomas Ritter

**Affiliations:** 1 College of Medicine, Nursing and Health Sciences, School of Medicine, Regenerative Medicine Institute, National University of Ireland, Galway, Ireland; 2 Institute for Multiple-Sclerosis Research, Department of Neuroimmunology, University Medicine, Göttingen, Germany; University of Pittsburgh, United States of America

## Abstract

Adult mesenchymal stem cells (MSCs) are non-hematopoietic cells with multi-lineage potential which makes them attractive targets for regenerative medicine applications. However, to date, therapeutic success of MSC-therapy is limited and the genetic modification of MSCs using viral vectors is one option to improve their therapeutic potential. Ex-vivo genetic modification of MSCs using recombinant adenovirus (Ad) could be promising to reduce undesired immune responses as Ad will be removed before cell/tissue transplantation. In this regard, we investigated whether Ad-modification of MSCs alters their immunological properties *in vitro* and *in vivo*. We found that Ad-transduction of MSCs does not lead to up-regulation of major histocompatibility complex class I and II and co-stimulatory molecules CD80 and CD86. Moreover, Ad-transduction caused no significant changes in terms of pro-inflammatory cytokine expression, chemokine and chemokine receptor and Toll-like receptor expression. In addition, Ad-modification of MSCs had no affect on their ability to suppress T cell proliferation *in vitro*. *In vivo* injection of Ad-transduced MSCs did not change the frequency of various immune cell populations (antigen presenting cells, T helper and cytotoxic T cells, natural killer and natural killer T cells) neither in the blood nor in tissues. Our results indicate that Ad-modification has no major influence on the immunological properties of MSCs and therefore can be considered as a suitable gene vector for therapeutic applications of MSCs.

## Introduction

Adult mesenchymal stem cells or stromal stem cells (MSCs) are non-hematopoietic cells with multi-lineage potential [1]–[3]. They can be isolated from bone marrow (BM) and various other sources such as umbilical cord blood or adipose tissue and have the capacity to extensively proliferate *in vitro.* Their capacity to differentiate into various cell lineages (e.g. osteocytes, chondrocytes, adipocytes) and their *in vitro* proliferative potential makes them attractive targets for regenerative medicine applications [4]–[6]. To date, a number of studies have shown that MSCs can migrate and successfully engraft in damaged organs and tissues [7], [8]. Emerging evidence suggests that chemokine receptors and their ligands play an important role in the homing of these cells to sites of injury or infection [9]. However therapeutic success of MSC therapy has been limited and the genetic modification of MSCs is one option to improve their therapeutic potential [10]–[12]. Numerous pre-clinical studies have shown beneficial effects when gene-modified MSCs were applied in various disease models (for review see [12], [13]).

Most pre-clinical and clinical applications of gene therapy have utilized virus-based transfer of genetic material due to high transduction efficiency, cell tropism and levels of transgene expression, however, adverse immune reactions against the gene therapy vehicle and transduced cells or transgene products has raised serious concern [14]. *Ex-vivo* genetic modification of cells or tissues using recombinant adenovirus (Ad) could be one option to reduce undesired immune responses of direct Ad-injection as unbound Ad will be removed before cell/tissue transplantation. However, Toll-like receptor (TLR) triggering by viral capsid proteins or dsDNA may stimulate innate immune mechanisms and render transduced cells more susceptible to immune-mediated rejection *in vivo* [15]. Indeed, it has been shown previously that differentiated cells e.g. cardiomyocytes and pancreatic islet cells up-regulate various TLRs and chemokine receptors when transduced *ex-vivo* with an Ad-vector [16], [17].

Interestingly, it has been shown that MSCs are immunoprivileged both *in vitro* and *in vivo* due to their low expression profile of major histocompatibility complex (MHC) class I molecules and the lack of expression of MHC class II molecules. Moreover, numerous studies have shown that MSCs do not express co-stimulatory molecules necessary for full activation of T cells, namely, CD80, CD86 and CD40 and also secrete anti-inflammatory cytokines e.g. Transforming Growth Factor (TGF)-β and Interleukin (IL)-10. It is thought that because of these unique features, MSCs can evade immune-mediated elimination [18]–[21]. Interestingly, Chuang and colleagues have recently shown that transduction of MSCs using recombinant baculovirus only led to a mild up-regulation of immune response parameters which did not impair their *in vivo* persistence [22]. Recombinant Ad has been extensively used for the genetic modification of MSCs [23]–[26], however, the immune profile of adenovirally transduced MSCs is not known.

The aim of this study was therefore to investigate if genetic modification of BM-derived MSCs using recombinant Ad alters the expression profile of immunologically relevant parameters such as MHC class I and II, co-stimulatory molecules, pro-inflammatory cytokine expression, chemokine/chemokine receptors or toll-like receptors which, consequently, may lead to an increased risk of recognition by the host immune system. Finally we investigated if *ex-vivo* Ad-transduced MSCs increase the host immune response after systemic injection in rats.

## Materials and Methods

### Ethics statement

All procedures performed on animals were approved by the Animal Ethics Committee of the National University of Ireland, Galway and conducted under licence from the Department of Health, Ireland. In addition, animal care and management followed the Standard Operating Procedures of the Animal Facility at the National Centre for Biomedical Engineering Science, Galway, Ireland.

### Bone marrow-derived rat mesenchymal stem cell culture and expansion

Bone marrow cells were extracted from male Sprague-Dawley (CD, Harlan Laboratories, UK) rats (8–12 weeks old) as detailed elsewhere [27]. Briefly, the animals were euthanized by CO_2_ inhalation and bone marrow cells were obtained by flushing femurs and tibias with a mixture of alpha modified Eagle's medium/Ham's F12 nutrient mixture (αMEM-F12; both Sigma-Aldrich, Dublin, Ireland). This cell suspension was then washed once with Dulbecco's Phosphate Buffered Saline (DPBS [Invitrogen, Dun Laoghaire, Ireland]). The centrifuged cells were then transferred to T-175 flasks at a density of 9×10^5^ cells/cm^2^ and rat MSC medium (αMEM-F12; 10% fetal bovine serum [FBS; Sigma-Aldrich] with penicillin/streptomycin supplements [Invitrogen]) was added to a final volume of 30 ml. The cultures were maintained at 37°C, 5% CO_2_ and 90% humidity. On day 3 medium and non-adherent cells were removed and replaced with fresh rat MSC medium. The medium was changed every 3–4 days until confluency was nearly reached. At the end of culture, adherent cells were detached using 0.25% trypsin/1 mM EDTA (Sigma-Aldrich). MSCs between passage 4 and passage 8 (P4–P8) were used for subsequent transduction and transplantation experiments.

To assess the osteogenic and adipogenic potential of rat MSCs, cells were plated in 6 well plates at various densities; 30,000 cells/well (osteogenic) and 2×10^5^ cells/well (adipogenic) and treated with specific induction medias the following day. Differentiation induction medias were prepared as previously described [28]. Quantification of mineral deposition (osteogenesis) and lipid accumulation (adipogenesis) were performed as previously described [28] using Alizarin Red and Oil Red O assays, respectively.

### Isolation of rat splenic dendritic cells

Spleens were collected from CD rats and overlaid with cold PBS/EDTA solution. 10 ml of this solution was added to a petri-dish and the spleen was divided up. The spleen was squeezed firmly (using a syringe handle) to release the dendritic cells (DCs). The 10 ml from the petri-dish was further filtered (using a 40 µm Nylon cell strainer) and the DCs were collected in a 50 ml tube. This step was repeated four more times, each time with 10 ml PBS/EDTA solution, giving a total cell suspension of 50 ml. Cells were centrifuged at 300 g for 8 mins at 4°C. Supernatant was removed and the cell pellet was re-suspended in 35 ml of cold PBS/EDTA solution and centrifuged again at 300 g for 8 mins at 4°C. Following centrifugation, the supernatant was removed and the cell pellet was re-suspended in 20 ml cold PBS/EDTA solution. The cell suspension was then divided into four tubes, each containing 5 ml. The 5 ml cell suspensions were then underlaid with 2 ml of Nycodenz (Progen Biotechnik, Heidelberg, Germany) solution. For the formation of the density gradient, the tubes were centrifuged at 300 g for 15 mins at 20°C. After centrifugation, the DCs were contained within a visible white ring. The DCs were then collected and washed twice with 15 ml of cold PBS/EDTA solution and centrifuged at 500 g for 5 mins at 4°C. The DCs were then re-suspended in 1 ml of warm RPMI solution and counted using a haemocytometer. Finally, the DCs were seeded in 6 well plates (approximately 1×10^6^ cells/well) and incubated at 37°C, 5% CO_2_.

### RAW 264.7 cell culture

The mouse monocytic cell line RAW 264.7 (American Type Culture Collection (ATCC), Middlesex, UK) was used as a positive control for secretion and detection of IL-1β after Ad-transduction. Cells were seeded in culture medium containing DMEM supplemented with 10% FCS, 2 mM glutamine, 1.5 g/l sodium bicarbonate, 4.5 g/l glucose and 1 mM sodium pyruvate (all Sigma-Aldrich) and incubated at 37°C, 5% CO_2_. Cells were passaged every 2/3 days and were not allowed to grow beyond 80% confluency.

### Genetic modification of mesenchymal stem cells

First generation E1/E3-deleted serotype 5 adenovirus encoding GFP (Ad.GFP) under the control of the cytomegalovirus (CMV) immediate early promoter was generated and purified as described elsewhere [29]. Ad.GFP transduction of P4–P8 MSCs was performed as described [23]. Briefly, MSCs were seeded in 6 well plates at a density of 100,000 cells/well in 2 ml of MSC medium for 24 h. Medium was then removed and Ad.GFP (or Ad.β-Gal [30] if GFP fluorescence interfered with other components of the experiment) was added at a multiplicity of infection (MOI) of 100 to the cells followed by spin centrifugation for 90 min at 2,000 g at 37°C. The medium was removed and fresh MSC medium added to the cells. Light and fluorescent microscopy was performed 24 h after transduction. Cells were detached with 0.25% trypsin/1 mM EDTA at 37°C and centrifuged at 400 g for 5 mins. After washing, cell pellets were resuspended in FACS buffer. The percentage of cells expressing GFP was then measured using a FACS Aria (BD Biosciences). For particular experiments, where indicated, MSCs were transfected with pNFκB-d2EGFP (a reporter plasmid that permits expression of GFP under control of the NFκB transcription factor binding sites [Clontech, Saint-Germain-en-Laye, France]) using TurboFect™ *in vitro* Transfection Reagent (Fermentas, York, UK). Also, in these experiments, transduction of pNFkB-d2EGFP transfected cells with Ad.β-Gal (MOI 100) was carried out so as not to interfere with GFP expression of transfected cells.

### Stimulation of mesenchymal stem cells with dendritic cell-conditioned medium

Bone marrow derived dendritic cells (BMDCs) were stimulated on day 9 of culture with 1 µg/ml of lipopolysaccharide (LPS) in a total volume of 3 ml per well of a 6 well plate. 24 h later supernatant was harvested, filter sterilised (0.2 µm) and added to either untransduced or Ad.GFP transduced MSCs in 6 well plates in a 1∶1 ratio with MSC medium in a final volume of 1.5 ml per well. Prior to addition to MSCs, samples of LPS-stimulated BMDC-conditioned medium were taken and screened for a panel of proinflammatory cytokines. It was found that the LPS-stimulated BMDCs produced higher levels of IL-1α, IL-1β, IFNγ, TNFα and IL-6 compared to unstimulated BMDCs (data not shown). Untransduced and Ad.GFP transduced MSCs were incubated for 24 h with the BMDC-conditioned medium. Cells were then detached using 0.25% trypsin/1 mM EDTA (Sigma-Aldrich), collected and total RNA was extracted with Trizol-LS reagent according to manufacturer's recommendations (Invitrogen).

### Real-Time RT-PCR

Table 1 contains the complete list of primer sequences used in this study. Total RNA was extracted from Ad.GFP-transduced and untransduced MSCs (1×10^5^ cells/well of 6 well plates) with Trizol-LS reagent according to manufacturer's recommendations (Invitrogen). cDNA was synthesized using RevertAid™ H Minus Reverse Transcriptase (Fermentas) with random primers. Two step qRT-PCR was performed to determine the mRNA expression levels of the chemokines, chemokine receptors and proinflammatory cytokines listed in Table 1 by comparing Ct values with that of the house-keeping gene β-actin. All quantitative real-time PCR was performed according to the standard program on the ABI one-step machine (Applied Biosystems, Warrington, UK).

**Table 1:** Chemokine, chemokine receptor, TLR and pro-inflammatory cytokine primer sequences used for quantitative real-time reverse transcription PCR.

Gene	Forward	Reverse	PROBE
β-actin	GTACAACCTCCTTGCAGCTCCT	TTGTCGACGACGAGCGC	CGCCACCAGTTCGCCATGGAT
GFP	GCCACAAGTTCAGCGTGTCC	GCTTCATGTGGTCGGGGTAC	CGGCAAGCTGACCCTGAA
IFNγ	AACAGTAAAGCAAAAAAGGATGCATT	TTCATTGACAGCTTTGTGCTGG	CGCCAAGTTCGAGGTGAACAACCC
IL-1β	AACAGCAATGGTCGGGACATA	CATTAGGAATAGTGCAGCCATCTTTA	TTGACTTCACCATGGAACCCGTGTCTT
IL-6	TCAACTCCATCTGCCCTTCAG	AAGGCAACTGGCTGGAAGTCT	AACAGCTATGAAGTTTCTCTCCGCA
CCL2	GTTGTTCACAGTTGCTGCCTGTAG	AGTGAATGAGTAGCAGCAGGTGAGT	TGCTGTCTCAGCCAGATGCAGTTAATGC
CCL4	TTCTGCGATTCAGTGCTGTCA	AATCTTCCGGGAGGTGTAAGAGA	CACCAATAGGCTCTGACCCTCCCACTTC
CCL5	CAACCTTGCAGTCGTCTTTGTC	GATGTATTCTTGAACCCACTTCTTCTC	AGGAACCGCCAAGTGTGTGCCAAC
CCR2	CACTTAGACCAGGCCATGCA	ACTTCTCACCAACAAAGGCATAAAT	TGACAGAGACTCTTGGAATGACACACTGCTG
CCR5	GTTCTCCTGTGGACCGGGTATAG	ATTGTCAAACGCTTCTGCAAAC	AGCTTACACGATCAGGATTGACTTGC
CCR7	CTCTTCAAGGACTTGGGCTG	GGGGAGAAGGTGGTGGTAGT	ACGGCTCCGGCAGTGGTCTTCC
CXCL1	TTCCTGGGAGTCTGCTGCTT	CACACAGATACTCTCTTGTAGGACTTCA	CATGGGTTGTGGAAGGTGTGGGC
CXCL2	GTTGAGGTACAGGACCCCATGT	GAAGCCCCCTTGGTTCAGA	TCCAGGTCAGTTAGCCTTGCCTTTGTTCAG
CXCL9	TTGCCCCAAGCCCTAACTG	ACCCTTGCTGAATCTGGGTCTAC	CATCGCTACACTGAAGAACGGAGATCA
CXCL11	GGTTCCAGGCTTCGTTATGTT	AACTTCCTTGATTGCTGCCATT	CTGTCTTTGCATCGACCGCGGAGT
CXCL12	GTCAAACATCTGAAAATCCTCAACAC	GGTCAATGCACACTTGTCTGTTGT	ACTGTGCCCTTCAGATTGTTGCAAGGCT
CXCR1	TTCGCCATGAATTCCTCAAGA	GTGGTGAGAGACGTGCGAAAG	CTTGCTAACCTGGTTCACAAGGAGG
CXCR2	CTTGAGAAGTCCATGGCGAAA	GCTTTGGAAGCTACTGAGATTCTTG	TTCTGGCCAATAAAGGCATAGATGATGGG
CXCR3	AGCAGCCAAGCCATGTACCTT	TAGGGAGATGTGCTGTTTTCCA	AGGTCAGTGAACGTCAAGTGCTAGATGCCTC
CXCR4	GGAGGTCATCAAGCAAGGATGT	GGGTTCAGGCAACAGTGGAA	TTCGAGAGCGTCGTGCACAAGTGG
CX3CR1	CTGGCACTTCCTGCAGAAGTC	CATACTCAAAGTTCTCTAGATCCAATTCC	AGCTGCTCAGGACCTCACCATGC
CX3CL1	GCTCATCCACTATCAACTGAACCA	TCCTTTGGGTCAGCACAGAAG	CAAGCGCGCCATCATCCTGGA
TLR1	AGTCAAGTCTTTCTCAATTTCA	TGACAACTTGATGTATCGACAA	AACTTCAGAAGATTCCATTATTCTGA
TLR2	AGAACTAAGAGATACTAACTTG	ACAGCTTCAGGAGTTCATTAAA	TTTCTGAACTGTCTGTAGACGAAAT
TLR3	ATAACGCATCACCTACTGAAA	AATCTGGAATATTCTGGAGAAAA	CCGCTGTGCAGAAGATTCAAGGT
TLR4	CCTGAAGATCTTAAGAAGCTAT	CCTTGTCTTCAATTGTCTCAAT	TTCACCAATTTCTCACAACTTCAGT
TLR5	CAAGACCGAACATTCAGATTA	ACCATCTGTACACTTGGAATAA	TACAAACCTTAGATCTCCGTGACAAT
TLR6	TGTCTCTCACAATCAGTTACAA	AGCTTCCTCAAGTTGCCAAAT	TCTCTTGCTGTCCTATGGTGAACT
TLR7	TCAGCCACAACCAGCTGACAA	AATTGCAAAGCATCTTCTAGAAA	CCTGCGAGATTGGCCAACTGTT
TLR8	TGCCAAGAGCTGGAACTTTAA	ATAATCACATCCATGTTCTCAT	TCTACTTGGCCTTGCAGAGGCTA
TLR9	CTGGACCTGTCCTATAACAA	ACAGATTGGCCAGAAAACTGA	ACCTGTACCATTCGAAATCGTTCA
TLR10	TATTATAAGCTGCACTGAGAAA	TGTAATCATAATTGTCACGGAAA	TATAAGTCCATCTTTGTGCTGTCC

### IL-1β ELISA

Cell culture supernatants were collected from Ad.GFP transduced/untransduced MSCs and Ad.GFP transduced, LPS stimulated and untransduced RAW 264.7 cells 48 hr post-transduction/stimulation. The concentration of IL-1β in the media was measured using a rat IL-1β Tissue Culture ELISA Ready-SET-Go!® (eBioscience) detection kit according to manufacturer's instructions.

### Stimulation of mesenchymal stem cells with TLR Ligands

2×10^5^ MSCs/well were seeded in 6 well plates containing 3 ml of serum-containing medium. After overnight incubation at 37°C, 5% CO_2_, serum-containing medium was removed and the appropriate TLR ligand dilutions, in serum-free medium, were added (Pam3CSK4 for TLR-2 stimulation (1 µg/ml, Invivogen, Toulouse, France) and ODN2395 for TLR-9 stimulation (5 µM, Invivogen)).

### Western Blot Analysis

Ad.GFP-transduced and untransduced MSCs were harvested and solubilised in protein lysis buffer complete with protease inhibitors. 30 µg of total protein for each sample was separated by 12% SDS-PAGE and electroblotted onto a nitrocellulose membrane. CX3CR1 was detected using a rabbit polyclonal IgG antibody (1∶2000 dilution; eBioscience). Cellular α-tubulin levels were detected using a mouse anti-α-tubulin antibody (1∶1000 dilution; eBioscience). Membranes were then incubated with HRP-labelled secondary antibodies (1∶1000 dilution; eBioscience) and detection was performed using SuperSignal West Pico chemiluminescent substrate (Thermo Scientific, Cheshire, UK).

### Transwell-based chemotaxis assay

Cell migration assays were performed using Corning (Fisher-Scientific, Loughborough, UK) Transwell inserts with 8 µm membrane pores placed in a 24 well plate. Prior to use, the membrane inserts were rehydrated with basal media (comprising αMEM and penicillin/streptomycin only) for 1 h. The migratory capacity of both Ad.GFP transduced or untransduced MSCs was assessed in response to rat fractalkine (20 µg, Cat. No. 400-26, Peprotech, Rocky Hill, NJ, USA) which was diluted in basal media (30 ng/µl) and added to the lower compartments of the wells. Basal media alone or basal media+FBS served as negative and positive controls, respectively. Once rehydrated, the membrane inserts were transferred to the 24 well plate and 1×10^5^ Ad.GFP transduced or untransduced MSCs in 0.1 ml basal media was added to the upper compartments. The cells were then incubated for 18–20 h at 37°C, 5% CO_2_. Inserts were removed, wiped off on the upper side, air-dried and stained with haemotoxylin for 3 mins. They were then washed twice in distilled water and air-dried at room temperature for 15 mins. Membranes were excised using a scalpel, placed on microscope slides and examined at 20× magnification.

### T-cell proliferation assay

T cells were isolated from lymph nodes of CD rats, processed by disruption of the tissue with a syringe plunger into a single cell suspension, washed with 0.1% BSA/PBS and stained in prewarmed (37°C) 10 µM carboxyfluorescein diacetate succinimidyl ester (CFSE)/PBS staining solution (CellTrace™ CFSE Cell Proliferation Kit, Invitrogen, Carlsbad, CA) at a concentration of 2×10^7^ cells/ml. Cells were incubated for 6 min/37°C protected from light and the reaction was stopped by adding 5 volumes of ice-cold medium containing 10% FBS. T cells were washed three times with final culture medium to remove all traces of unbound CFSE. 2×10^5^ CFSE-stained T cells were stimulated in 96-well plates with an equal amount of anti-rCD3/anti-rCD28-labelled beads in MLC medium (2% heat-inactivated rat serum, 10% FBS, 50 µM β-mercaptoethanol in RPMI 1690 (Sigma Aldrich)). Varying amounts of MSCs were added. T cells were harvested after 4 days and CFSE fluorescence of cells was analysed using a FACS Canto (BD Biosciences).

### Cytofluorimetric analysis

The following monoclonal antibodies were used for phenotypic characterisation of MSCs and immune cell subsets: anti-rat MHC class I-FITC (OX-18, Cat. No. MCA5IFT, AbD Serotec, Kidlington, UK), anti-rat CD29-FITC (Ha2/5, Cat. No. 555005), anti-rat CD44H-FITC (OX-49, Cat.No. 550974), anti-rat CD45RA-PE (OX-1, Cat. No. 554878), anti-rat CD71-PE (OX-26, Cat. No. 554891), anti-rat CD73-PE (5F/B9, Cat. No. 551124), anti-rat CD90-FITC (OX-7, Cat. No. 551401) (all from BD Biosciences, San Jose, CA), anti-rat MHC class II-FITC (OX-6, Cat. No. 205305), anti-rat CD3-FITC (1F4, Cat. No. 201403), anti-rat CD4-APC (W3/25, Cat. No. 201509), anti-rat CD8-PE (OX-8, Cat. No 201706), anti-rat CD11b/c-APC (OX-42, Cat. No. 201809), anti-rat CD25-FITC (OX-39, Cat. No. 202103), anti-rat CD80-PE (3H5, Cat. No. 200205), anti-rat CD86-PE (24F, Cat. No. 200308), anti-rat CD161-AF647 (10/78, Cat. No. 203110) (all from BioLegend, San Diego, CA, USA) and anti-rat HIS36-PE (HIS36, Cat. No. 12-0060-82, eBioscience, Hatfield, UK). For staining, cells were washed with FACS buffer (DPBS containing 2% FBS and 0.1% NaN_3_, both from Sigma-Aldrich). mAbs were diluted in 50 µl FACS buffer, added to the cells and incubated for 30–45 mins at 4°C. Following incubation, unbound antibody was removed by washing the cells twice with FACS buffer. Cells were then resuspended in FACS buffer and analysed using a FACS Canto (BD Biosciences). Data were analysed and compensated using FlowJo (Tree Star, Inc., Ashland, OR, USA) or Diva software (BD Biosciences).

### *In vivo* experiments

Male Sprague Dawley (CD) rats used in this study were obtained from Harlan Laboratories UK. All animals used were between 8 and 12 weeks of age. Animals were briefly anesthetized with isofluorane for intravenous injection with either 2×10^6^ untransduced (n = 3) or 2×10^6^ Ad.GFP transduced MSCs (n = 4). Blood was taken at various time points and animals were sacrificed at day 14 post-injection by CO_2_-inhalation. Organs were harvested and prepared for flow cytometry. Briefly, single cell suspensions from lungs of treated animals were prepared by digesting the tissue with 5% w/v Collagenase D (Sigma-Aldrich) in RPMI containing 25 mM HEPES (Lonza, Basel, Switzerland) plus 1% FCS (Sigma-Aldrich) at 37°C for 2 h. The digested tissue was then poured into a 100 µM cell strainer and disrupted using a syringe plunger. Cell suspensions were transferred to 15 ml tubes, centrifuged at 400 g for 5 min and washed with PBS. Spleens from treated animals were disrupted using a syringe plunger. Cell suspensions were transferred to 15 ml tubes and centrifuged at 400 g for 5 min. Cell pellets were resuspended in ACK cell lysis buffer (containing 0.15 M ammonium chloride, 10 mM potassium bicarbonate and 0.1 mM EDTA (all Sigma-Aldrich) dissolved in distilled water) and incubated at room temperature for 10 min. Cells were centrifuged at 400 g for 5 min and washed with PBS. PBMCs were isolated from whole blood by incubating with ACK buffer for 10 min at room temperature. Cell suspensions were centrifuged at 400 g for 5 min and washed with PBS. Cell suspensions from lungs, spleens and blood were subsequently used for multicolour flow cytometric analysis.

### Statistical Analysis

Significance was assessed by student's *t* test or non-parametric Mann-Whitney test. Differences were considered significant if p≤0.05.

## Results

### *In vitro* responses

#### Characterization of rat mesenchymal stem cells

**Figure 1:**
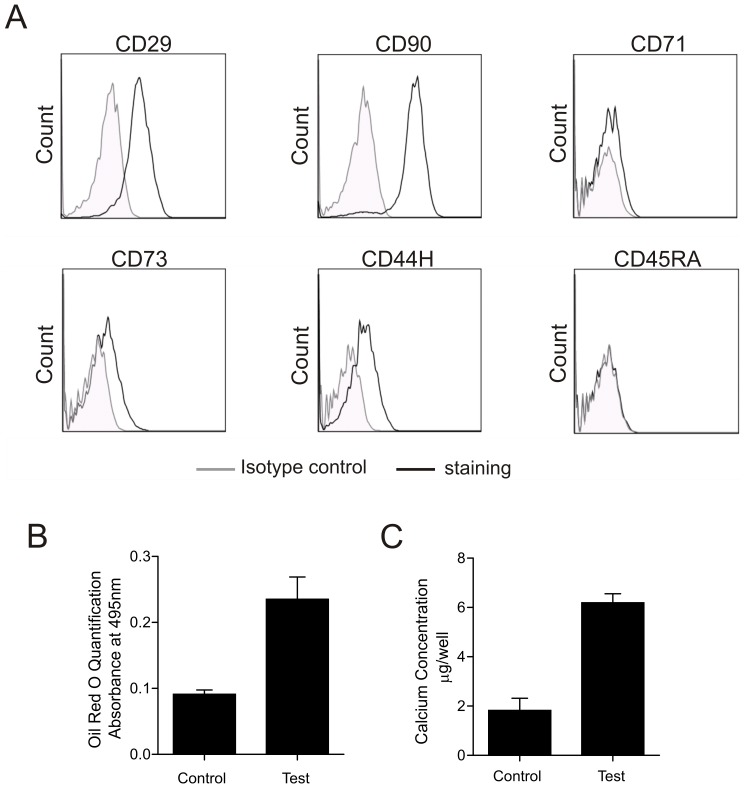
Cell surface characterization and differentiation potential of MSCs. (A) Cell surface expression of various MSC markers were detected by staining with specific monoclonal antibodies and analysed by flow cytometry. MSCs are CD29, CD73, CD90 and CD44H positive, and CD45RA, CD71 low or negative. Shown are FACS histograms of CD MSCs stained with antibodies against surface markers as indicated or with appropriate isotype controls in triplicates. MSCs were tested for their potential to differentiate into (B) adipocytes and (C) osteocytes by incubating the cells with specific formulations. Results shown are representative data of 3 separate isolations.

Rat MSCs were isolated from bone marrow (BM) of CD rats, cultured and subsequently characterized for the expression of relevant cell surface markers and their potential to differentiate into adipocytes and osteocytes. Flow cytometric analysis showed that MSCs express the cell surface markers CD29, CD73, CD90, and CD44H and lowly express or are negative for CD45RA and CD71 (Figure 1A). MSCs can differentiate along the adipogenic and osteogenic lineages as measured by Oil Red O quantification and calcium concentration (Figure 1B and C, respectively). In summary our results show that the cells isolated from BM of rats are truly MSCs which are being used in all subsequent experiments.

**Figure 2:**
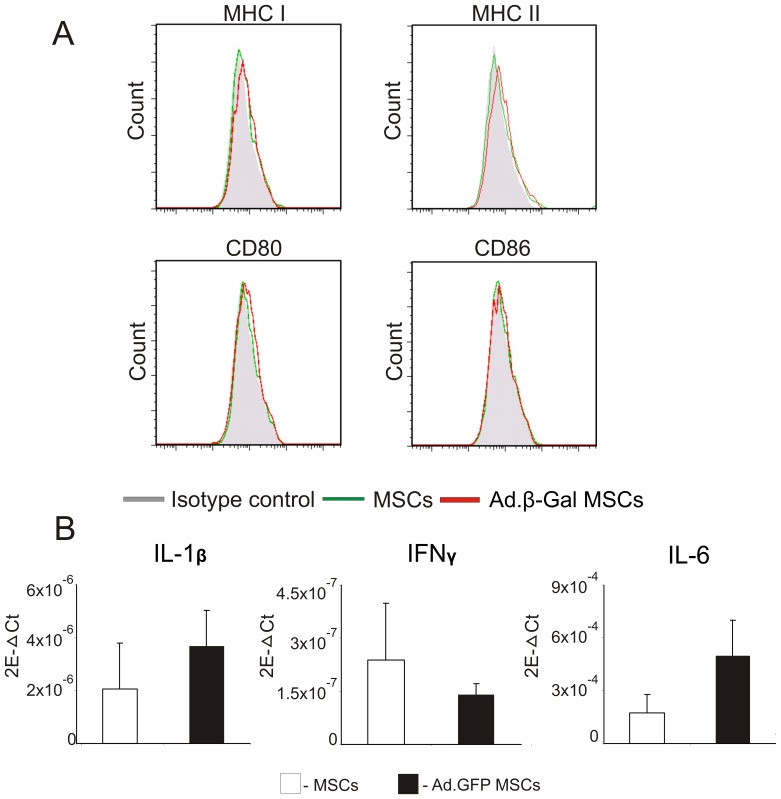
Immunophenotyping and pro-inflammatory cytokine expression of untransduced and Ad-transduced MSCs. (A) Untransduced and Ad-transduced MSCs were stained for expression of the immunologically relevant markers MHC class I/II, CD80 and CD86 with specific antibodies and analysed by flow cytometry. (B) MSCs were transduced with Ad.GFP and after 24 h cells were collected and subjected to mRNA isolation and cDNA synthesis. RT-PCR analysis showing mRNA expression levels of IL-1β, IFN-γ and IL-6 from untransduced and Ad-transduced MSCs. Data shown are means ±SD of three independent experiments. 2E-ΔCT = 2^−ΔCT→^ number of copies of gene of interest relative to the number of copes of the internal control gene, β-actin.

MSCs can be readily transduced with adenoviral vectors at a MOI of 100 followed by spin centrifugation. The mean transduction efficiency in this study was 66% (n = 3, representative gating shown in figure S1A) as determined by fluorescence activated cell sorting (FACS) analysis. Due to limited transduction efficiencies, Ad.GFP MSCs were FACS sorted into GFP positive (+) and GFP negative (−) fractions (n = 3, figure S1A). From the two distinct populations, we proceeded to isolate RNA, synthesise cDNA and perform RT-PCR analysis (as described in Materials and Methods) to analyse the mRNA expression levels of the chemokines, chemokine receptors and TLRs listed in Table 1. Consistently, we observed non-significant differences in expression levels between the two cell populations (figure S1B) and, as a result, a transduction efficiency of 66% was deemed adequate for subsequent experiments. Moreover, it was decided that while an increased MOI could be used and thus improve transduction efficiency, we found that this leads to a substantial increase in cytotoxicity (unpublished observations) and therefore would compromise the viability of the Ad.GFP transduced MSCs *in vivo*. In order to understand if Ad-transduction of MSCs alters the immunologically relevant cell surface marker expression profile, transduction of MSCs using an adenovirus encoding for the reporter gene β-Galactosidase (Ad.β-Gal) at a MOI of 100 followed by spin centrifugation was carried out. After 24 h MSCs were collected and analysed for expression of specific cell surface markers by flow cytometry. As shown in Figure 2A, transduction of MSCs with Ad.β-Gal did not alter the cell surface expression profile of immunologically relevant cell surface markers. Neither an increase in MHC class I and II cell surface expression nor an increase in expression of co-stimulatory molecules CD80 or CD86 was observed upon transduction with Ad-vectors (n = 3).

We also sought to elucidate whether Ad-modification of MSCs may cause the cells to switch to a more pro-inflammatory phenotype. Therefore, we analysed mRNA expression levels of a number of pro-inflammatory cytokines, namely, Interleukin (IL)-1β, IL-6 and Interferon (IFN)-γ (Figure 2B; primer sequences shown in Table 1). We found that mRNA expression levels of IL-1β and IL-6 were slightly but not significantly up-regulated in Ad-transduced MSCs compared to untransduced MSCs and expression levels were generally quite low. Due to its importance in the early inflammatory response to a range of different viruses, we sought to confirm our data on IL-1β mRNA expression at the protein level. We therefore examined the levels of IL-1β in cell culture supernatants of untransduced or Ad-transduced MSCs by specific ELISA. A mouse monocytic cell line (RAW 264.7) which can be readily transduced with Ad-vectors served as a positive control. IL-1β protein expression levels of both untransduced and Ad-transduced MSCs fell below the detection limits of the kit (sensitivity = ≥2.4 pg/ml, data not shown) thereby confirming our data on mRNA expression levels. In contrast, both control and Ad-transduced RAW cells secrete high levels of IL-1β (≥2.5 µg/ml) into the culture supernatant following stimulation with LPS (data not shown). These data indicate that untransduced MSCs do not secrete IL-1β in detectable amounts and – more importantly – this does not change upon genetic modification with Ad-vectors.

#### Comparison of chemokine/chemokine receptor mRNA expression profile of untransduced and Ad-transduced rat mesenchymal stem cells

**Figure 3:**
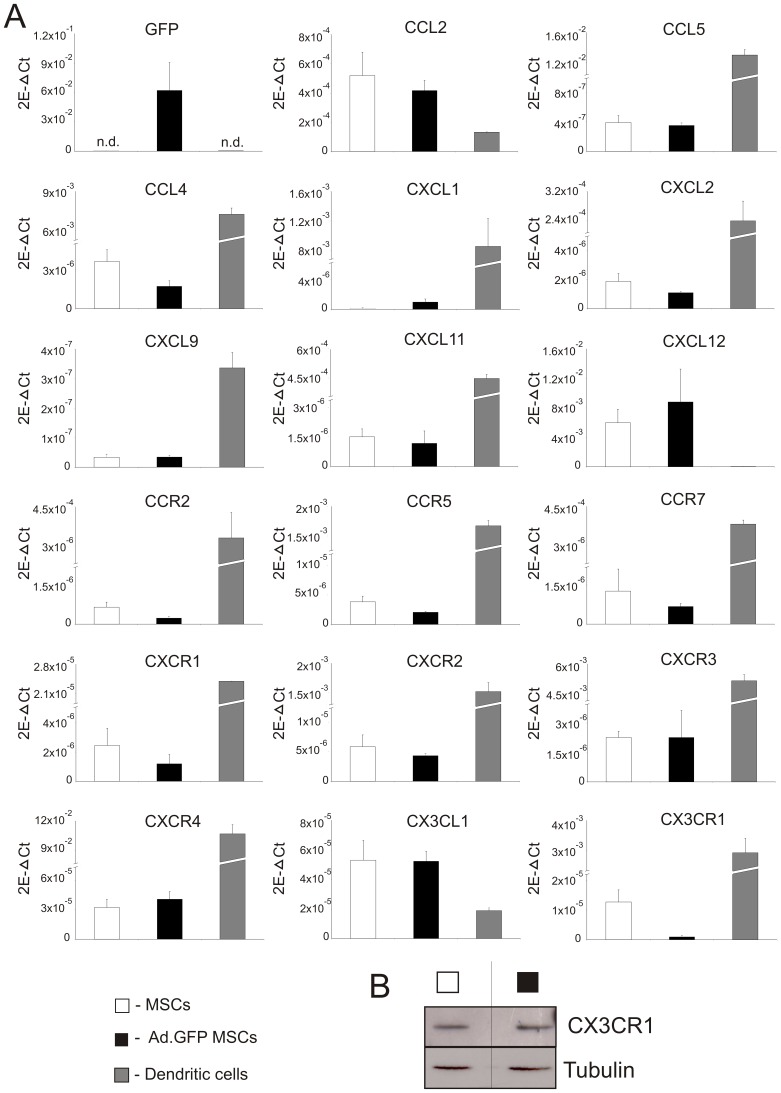
Chemokine/chemokine receptor mRNA expression profile of untransduced and Ad-transduced MSCs. (A) RT-PCR analysis showing mRNA expression levels of a panel of chemokines and chemokine receptors from untransduced and Ad-transduced MSCs. Freshly isolated DCs served as a positive control. Expression levels were normalized to the mRNA expression level of the constitutively expressed housekeeping gene β-actin. Data shown are means ±SD of four independent experiments. (**B**): CX3CR1 protein expression analysis by Western blot, 48 h post-transduction or no transduction, untransduced vs. Ad-transduced MSCs. Tubulin expression served as control. 2E-ΔCT = 2^−ΔCT→^ number of copies of gene of interest relative to the number of copies of the internal control gene, β-actin.

The enhanced secretion of chemokines upon genetic modification of MSCs may render the cells more susceptible for immune recognition by the host organism. Therefore we analysed if the chemokine and chemokine receptor mRNA expression profile of untransduced MSCs was altered after Ad-transduction. mRNA was isolated from either untransduced or Ad-transduced MSCs and subjected to real time RT-PCR analysis using specific primers and probes (sequences for all chemokines and chemokine receptors are shown in Table 1). Rat professional antigen presenting cells (dendritic cells, DCs) known to express multiple chemokine/chemokine receptors served as a positive control. As shown in Figure 3A, the majority of chemokines and chemokine receptors analysed in this study were expressed by both MSCs and rDCs (n = 4). Interestingly, CCL2 (MCP-1), CXCL12 (SDF-1α) and CX3CL1 (fractalkine) were the only chemokine mRNAs expressed at higher levels in MSCs compared to DCs which is in agreement with a number of other studies [17], [31], [32]. For all other studied molecules, however, mRNA expression levels were between factor 10 and 1×10^5^ times lower in MSCs compared to DCs. Moreover, genetic modification of MSCs using Ad-vectors did not lead to a significant up- or down-regulation of chemokine or chemokine receptor mRNA expression (Figure 3A). It was, however, interesting to note that in Ad-transduced MSCs mRNA levels of CX3CR1 (the receptor for fractalkine) were profoundly reduced compared to untransduced MSCs. To investigate if this has any influence on the migratory capacity of Ad-transduced MSCs a cell migration experiment was set up by seeding either untransduced or Ad-transduced MSCs in transwells and allowing them to migrate in response to CX3CL1 (fractalkine) over a period of 18–20 hr. In general, we observed low levels of MSC migration in response to the chemoattractant and no difference in the migratory capacity of untransduced vs. Ad-transduced MSCs (data not shown). Moreover, Western blot analysis of CX3CR1 protein expression did not show any difference between untransduced and Ad-transduced MSCs (Fig. 3B). In summary these data indicate that Ad-transduction of MSCs does not induce major changes in their chemokine/chemokine receptor expression profile which also does not seem to affect their migration potential.

Furthermore, as in a transplant setting or disease model the infused MSCs could potentially encounter recipient antigen presenting cells such as DCs in an activated state due to an ongoing inflammatory response, we were interested to investigate what effect inflammatory stimuli may have on chemokine receptor expression by both the untransduced and Ad-transduced MSCs. To mimic this we set up an *in vitro* assay in which BMDCs were stimulated with LPS for 24 h, after which time the supernatant was removed and added to either the untransduced or Ad.GFP transduced MSCs. We then performed RT-PCR analysis on a number of chemokine receptors and, as shown in figure S2, we saw no significant changes in mRNA expression levels of CCR2, CCR5, CXCR3, CXCR4 or CX3CR1 (containing members of the three main sub-classes of chemokine receptors) even after stimulation with potent inflammatory stimuli.

#### Comparison of Toll-Like Receptor mRNA expression profile of untransduced and Ad-transduced rat mesenchymal stem cells

**Figure 4:**
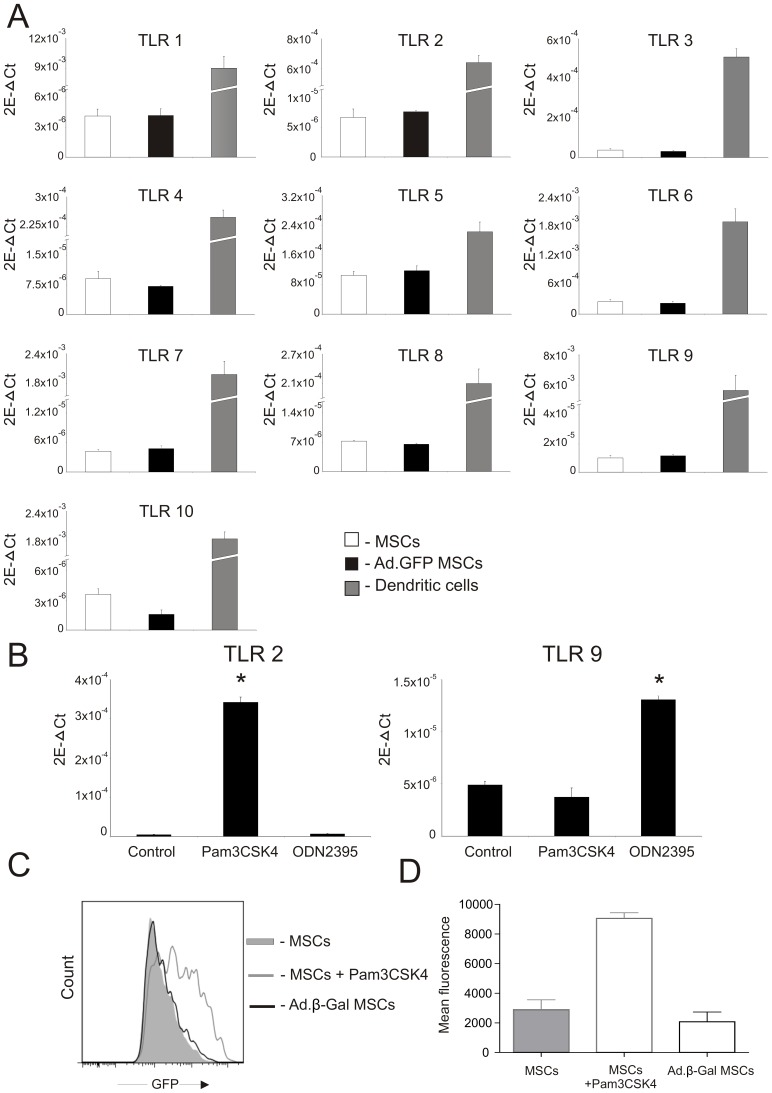
TLR mRNA expression profile of untransduced and Ad-transduced MSCs. (A) RT-PCR analysis showing mRNA expression levels of TLRs 1–10 from untransduced and Ad-transduced MSCs. rDCs served as a positive control. Expression levels were normalized to the mRNA expression level of the constitutively expressed housekeeping gene β-actin. Data shown are means ±SD of four independent experiments. (**B**) Functional activation of TLR 2 and 9 following ligand-specific stimulation. RT-PCR analysis showing mRNA expression levels of TLR 2 and 9 by MSCs following stimulation with Pam3CSK4 and ODN2395. *: p<0.05 compared to control. (**C**) FACS analysis of GFP-expressing MSCs following transfection with pNFκB-d2EGFP, and subsequent transduction with Ad.β-Gal (black line) or stimulation with Pam3CSK4 (grey line), respectively, compared to untreated MSCs (filled histogram). 2E-ΔCT = 2^−ΔCT→^ number of copies of gene of interest relative to the number of copies of the internal control gene, β-actin.

Next we analysed the TLR mRNA expression profile of MSCs 24 h after transduction with Ad-vectors. mRNA was isolated from either untransduced or Ad-transduced MSCs and subjected to real time RT-PCR analysis using specific primers and probes (sequences shown in Table 1). Rat DCs known to express high levels of TLRs served as a positive control [33]. As shown in Figure 4A, both MSCs and DCs express TLRs 1–10 at the mRNA level (n = 4). However, our results indicate that MSCs have much lower TLR mRNA expression levels compared to DCs. Importantly, we found that genetic modification of MSCs with Ad-vectors only resulted in negligible differences between the Ad-transduced and untransduced MSCs. Of particular interest was the finding that TLR2 and TLR9, both of which play a key role in Ad-induced inflammation [34], were expressed approximately 100 and 570 times, respectively, less in Ad-transduced MSCs compared to DCs.

Next we wanted to investigate if – despite very low mRNA expression levels and minor changes upon transduction with Ad-vectors - TLR2 and TLR9 are functionally active in MSCs. Moreover we sought to investigate if TLR mRNA expression levels would increase upon stimulation with either the TLR1/2 specific ligand Pam3CSK4 or the TLR9 specific ligand ODN2395 as it has been described before for professional antigen presenting cells [15]. As shown in Figure 4B, following stimulation with Pam3CSK4, TLR2 mRNA expression was significantly up-regulated on MSCs. Furthermore, while up-regulation of TLR2 was first detected after 3 hr (data not shown), this up-regulation was maintained over a period of 24 hr. This indicates that TLR2 signalling pathways are operational in MSCs upon TLR triggering. Interestingly, TLR2 mRNA was not up-regulated after Ad-mediated transduction of MSCs. In addition, TLR9 mRNA was also up-regulated upon triggering with the specific ligand ODN2395 (Fig. 4B). In summary these results indicate that TLRs 2 and 9 are functional in MSCs, however, no change in mRNA expression upon Ad-transduction is observed in MSCs.

It is known that TLR activation induces nuclear translocation of the key transcription factor NFκB from the cytosol, resulting in NFκB-dependent gene expression. To further analyse if TLR-mediated NFκB activation could occur in MSCs after Ad-transduction, MSCs were transfected with a plasmid which expresses GFP under the control of the NFκB transcription factor binding sites (pNFκB-d2EGFP). 24 hr after transfection MSCs were either stimulated with Pam3CSK4 or transduced with Ad.β-Gal (not to interfere with GFP expression induced from the plasmid), and analysed by flow cytometry for NFκB-mediated GFP expression. As shown in Figure 4C and D, stimulation with Pam3CSK4 led to a clear increase in GFP expression compared to untransduced MSCs, thereby indicating activation of the NFκB-pathway. However, in contrast, transduction of MSCs with Ad.β-Gal did not result in a significant change in GFP expression compared to untransduced MSCs (n = 3). This suggests that Ad-transduction of MSCs does not lead to proinflammatory signal transduction pathway activation.

#### Analysis of the capacity of Ad-transduced rat mesenchymal stem cells to inhibit T cell proliferation

**Figure 5:**
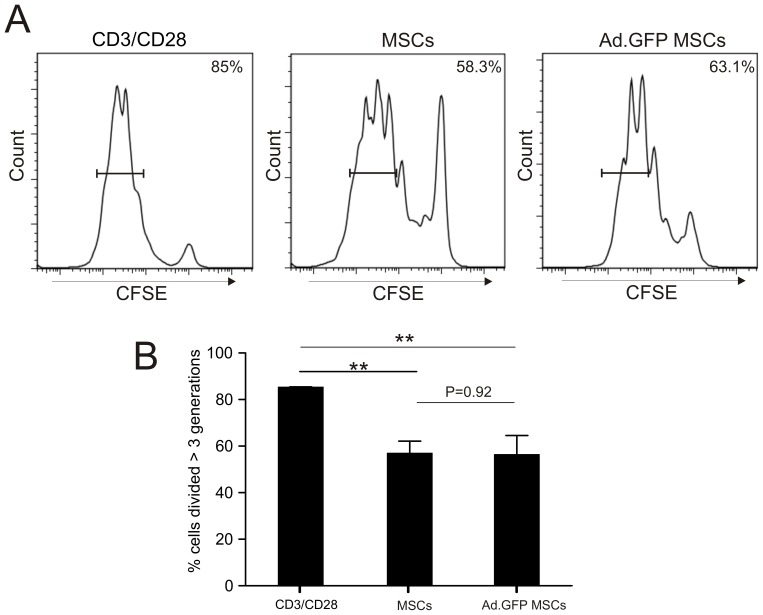
T-cell proliferation in the presence or absence of untransduced and Ad.GFP transduced MSCs. (A) Histograms showing percent proliferation of CFSE-labelled T cells that were polyclonally stimulated with anti-CD3/anti-CD28 beads in the absence or presence of either untransduced or Ad.GFP transduced MSCs (ratio of T cells to MSCs; 1∶250). (B) Bar chart showing the percentage of cells divided greater than three generations following polyclonal stimulation in the absence or presence of either untransduced or Ad.GFP transduced MSCs. CFSE fluorescence was analysed on day 4 after co-culture. Shown is a representative experiment with means of 4 replicates ± SD (**: p<0.01; student's T test).

Finally we investigated whether Ad-transduction of MSCs alters the capacity of MSCs to modulate T cell proliferation. For this, polyclonally activated T cells (stimulated with anti-CD3/anti-CD28 coated beads) were co-cultured with either untransduced or Ad.GFP-transduced MSCs. T cells were labelled with CFSE and after a 4-day co-culture with MSCs, collected and subjected to flow cytometric analysis. We found that co-culture of MSCs with polyclonally activated T cells significantly inhibited the proliferation of CFSE-labelled T cells and this was not altered upon genetic modification of MSCs using Ad-vectors (p<0.01, Figure 5A and B).

In summary, the results from this study so far suggest that negligible differences exist between untransduced and Ad-transduced MSCs in terms of TLR, chemokine/chemokine receptor, pro-inflammatory cytokine expression and the potential to inhibit T cell proliferation.

### *In vivo* responses

#### Investigating the in vivo immune response against Ad-transduced mesenchymal stem cells

**Figure 6:**
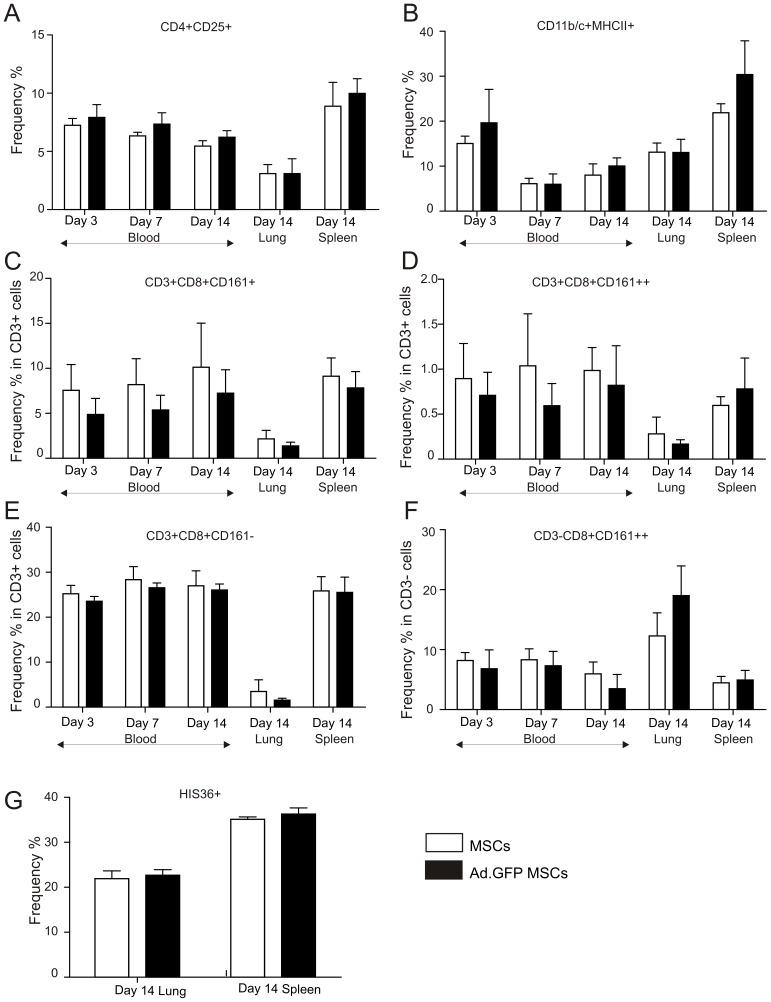
Analysis of immune cell populations after injection of untransduced or Ad-transduced MSCs. The percentages of (A) CD4+CD25+, (B) CD11b/c+MHCII+, (C) CD3+CD8+CD161+, (D) CD3+CD8+CD161++, (E) CD3+CD8+CD161− and (F) CD3−CD8+CD161++ cells in the PBMC population at days 3, 7 and 14, and also in the lung and spleen at day 14 post-injection of either 2×10^6^ Ad.GFP transduced MSCs (n = 4) or 2×10^6^ untransduced MSCs (n = 3) as measured by flow cytometry. (G) The percentage of HIS36+ cells in the lung and spleen at day 14 post-injection as measured by flow cytometry. Statistical analysis was performed using the non-parametric Mann-Whitney test. For all tested parameters, p values were >0.05.

In order to understand if genetic modification of MSCs using Ad-vectors may induce an inflammatory response *in vivo*, MSCs were transduced *ex-vivo* with Ad.GFP (MOI 100) and injected intravenously (i.v.) into syngeneic CD rats (2×10^6^ cells/animal (n = 4). Untransduced MSCs (2×10^6^ cells/animal) were injected as control (n = 3). Blood samples of treated animals were taken on days 3, 7 and on the day of sacrifice (day 14) and the frequency of several blood cell populations (antigen presenting cells, CD11b/c+MHCII+; activated T cells and cytotoxic T cells, CD4+CD25+ and CD3+CD8+CD161−; activated natural killer cells, CD3−CD8+CD161++ and natural killer T cells CD3+CD8+CD161+, CD3+CD8+CD161++) were analysed by flow cytometry. Moreover, spleens and lungs from animals treated with either untransduced or Ad.GFP-transduced MSCs were collected and analysed for the presence of the same immune cell populations as for blood. Additionally, we stained for HIS36 expression as evidence of tissue macrophage presence in lungs and spleens of treated animals on the day of sacrifice. No significant differences in the percentage and in the activation status of the different blood cell populations analysed was observed on days 3, 7 and 14 (Figure 6A–F and figure S3A for gating strategy) after injection of either untransduced or Ad.GFP-transduced MSCs. Moreover, similar results were observed when analysing the immune cell populations isolated from the lungs and spleens of both treatment groups (Figure 6A–G and figure S3B and C).

Altogether our *in vitro* and *in vivo* analysis indicates that Ad-modification of MSCs does not significantly alter their immunosuppressive profile which may have important implications for the use of Ad-transduced MSCs within a therapeutic setting.

## Discussion

In this manuscript we describe that Ad-modification has no major influence on the immunological properties of MSCs both *in vitro* and *in vivo* and therefore can be considered as a suitable gene vector for therapeutic applications of MSCs. This has not been described so far, however, it is an important finding in the field as more pre-clinical and clinical studies will utilise Ad-modification of MSCs to further enhance their therapeutic properties.

One of the key features regarding the low immunogenicity of MSCs is the very low expression of MHC class I and the absent expression of MHC class II molecules on their cell surface. Since MHC class I and II molecules play a pivotal role in the presentation of endogenously processed and phagocytosed antigens to CD8+ and CD4+ T cells, respectively, it was of interest to study if Ad-modification alters the cell surface expression profile of these molecules. Interestingly, we did not find notable differences in MHC class I/II and co-stimulatory receptor expression (CD80/CD86) after Ad-transduction of MSCs. (Figure 2A). However, this is in sharp contrast to the Ad-modification of professional antigen presenting cells such as DCs which up-regulate MHC molecules and co-stimulatory receptors upon Ad-transduction [35]. In this context it is a desired effect when Ad-modified DCs are being used, for example, as vaccine adjuvants for a more efficient generation of T-cell mediated immune responses *in vivo* [36]. Interestingly, transduction of MSCs with baculovirus seems to moderately up-regulate MHC class I expression, however, adverse immune stimulatory effects have not been reported [22]. In line with our previous observation on MHC class I and II expression we also found that the potential of MSCs to suppress T cell proliferation was not affected by Ad-modification (Figure 5A and B). Moreover, there was no significant change to the mRNA expression levels of key pro-inflammatory cytokines (IL-1β, IFN-γ, IL-6) upon Ad-transduction of MSCs (Figure 2B) which is again profoundly different when DCs are being modified with Ad-vectors [33]. These results suggest that transduction of MSCs with Ad-vectors neither impairs their immunosuppressive properties nor increases their immunogenicity.

There is increasing evidence that the interaction of viral vectors with a broad range of cell types leads to the immediate (and highly expressed) production of chemokines. This effect may render the cells more susceptible for immune recognition by the host organism. Chen and colleagues found that multiple chemokines and chemokine receptors were up-regulated in Ad-transduced murine cardiomyocytes compared to untransduced cardiomyocytes. Furthermore, they found that Ad-transduction induced a broader panel of chemokines and receptors than that induced by either ischemia or alloantigen [17]. Similarly, Zhang and co-workers reported significantly greater expression of a number of chemokines and chemokine receptors in Ad-transduced pancreatic islet cells compared to untransduced cells [16]. We found that, at least in terms of mRNA expression, there is little variation between untransduced and Ad-transduced MSCs with respect to the panel of chemokines/chemokine receptors that were analysed (Figure 3A). The only notable exception to this observation was in the case of CX3CR1 (fractalkine receptor), which showed a profound decrease in mRNA expression in Ad-transduced MSCs compared to untransduced cells (Figure 3A). However, we were unable to confirm this observation at the protein level by Western blot analysis (Figure 3B). Moreover, we also saw that the migratory capacity of MSCs in response to CX3CR1 was not influenced by Ad-transduction (data not shown).

It is well known that stimulation of TLRs with pathogens or specific ligands leads to the induction of pro-inflammatory cytokine production and alterations of immunomodulatory responses [15], [37]. We were interested in addressing if this could also be observed after Ad-transduction of MSCs. In particular the expression of TLR2 and TLR9 was monitored as these TLRs are thought to be involved in Ad-mediated inflammatory responses [15]. Our findings were that MSCs express very low levels of TLR mRNAs and Ad-transduction of MSCs did not lead to an increase in the mRNA expression of TLRs (Figure 4A and B), in contrast to what has been described previously upon Ad-transduction of DCs [33]. We further investigated if, despite low TLR-expression on MSCs, signalling via TLR receptors will occur after Ad-transduction. As TLR-signalling is pre-dominantly mediated by the transcription factor NF-κB we engineered MSCs to express a plasmid with NF-κB binding sites followed by a GFP-expression cassette. If TLR triggering induced NF-κB stimulation of GFP, expression would be induced and could subsequently be detected by flow cytometry. However, we found that MSCs do not respond after Ad-transduction whereas profound GFP expression was observed after triggering MSCs with specific TLR ligands (Figure 4C and D).

In summary our *in vitro* results indicate that Ad-transduction of MSCs does not significantly alter their immunosuppressive potential. However, only subtle changes in the innate immune expression profile of MSCs after Ad-transduction *in vitro* may have a profound stimulatory effect on various immune cell populations *in vivo*. To investigate this in more detail we injected either untransduced or Ad-transduced MSCs (i.v.) into syngeneic rats to specifically monitor the effect of Ad-modification only and analysed immune cell populations at different time points. It has been previously reported by others that after systemic administration into normal, non-injured animals, MSCs were found in the lungs, kidneys, spleens and bones for as long as 48 h [38]. Our own *in vivo* distribution data after injection of both CFSE-labeled untreated syngeneic and allogeneic MSCs confirm these results (Treacy, Nosov and Ritter, unpublished data). Based on the fact that the majority of these cells end up in the lung after systemic injection we carefully analysed if the induction of an immune response has occurred in this organ but also in the spleen (due to its critical role in immunomodulation) and in the blood of treated animals in the potential event of immune cell migration. Our analysis indicated that at day 3, day 7 and day 14 there was no significant difference in the frequency and the activation status of various immune cell populations (CD11b/c+MHCII+, CD4+CD25+, CD3+CD8+CD161+, CD3+CD8+CD161++, CD3+CD8+CD161−, CD3−CD8+CD161++) in the blood (Figure 6A–F) of treated animals. Moreover, similar results were observed when lungs and spleens of treated animals from both groups were collected and analysed at the end of the observation period (day 14). In addition, intramuscular (i.m.) injection of non-transduced or Ad-transduced MSCs in rats (n = 4) has been performed to analyze the local immune response. Eight days after i.m. injection animals were sacrificed and muscle tissues were collected and embedded in paraffin for tissue sectioning and immunostaining. The results were very similar to what we have observed after i.v. injection of non-transduced or Ad-transduced MSCs in that there was no difference in the distribution pattern of analysed immune cell populations (Treacy, Nosov and Ritter, unpublished data). These results indicate that Ad-transduced MSCs do not induce a detectable immune response after either systemic or local injection when compared to untransduced MSCs which may even allow repeated application of Ad-transduced MSCs if required.

In summary our data indicate that transduction of undifferentiated MSCs with Ad-vectors has no major influence on MSC-mediated immunosuppressive effects and therefore can be considered as a suitable gene vector for therapeutic applications of MSCs.

## Supporting Information

Figure S1 **mRNA expression levels of TLRs, chemokines and chemokine receptors post-FACS sorting into Ad.GFP+ and Ad.GFP− fractions.** (A) Representative dot plot and gating strategy of untransduced and Ad.GFP transduced MSCs as measured by flow cytometry. (B) Bar charts showing the mRNA expression levels of a representative selection of TLRs, chemokines and chemokine receptors post-FACS sorting into Ad.GFP+ and Ad.GFP− fractions, as measured by real-time RT-PCR. Shown is one representative experiment from two performed. 2E-ΔCT = 2^−ΔCT→^ number of copies of gene of interest relative to the number of copies of the internal control gene, β-actin.
(TIF)

Figure S2 **Chemokine receptor mRNA expression profile of untransduced and Ad-transduced MSCs following stimulation with BMDC-conditioned medium.** RT-PCR analysis showing mRNA expression levels of a panel of chemokine receptors from untransduced and Ad-transduced MSCs in the presence or absence of BMDC-conditioned medium. Data shown are means ±SD of two separate isolations. 2E-ΔCT = 2^−ΔCT→^ number of copies of gene of interest relative to the number of copies of the internal control gene, β-actin.
(TIF)

Figure S3 **Gating strategy used for the analysis of cell distribution in PBMCs, lungs and spleens.** Representative dot plots and gating strategy of (A) PBMCs, (B) lungs and (C) spleens from animals that each received an intravenous injection of 2×10^6^ Ad.GFP transduced MSCs (n = 4) or 2×10^6^ untransduced MSCs (n = 3).
(TIF)
