# Supplementary figures and images for: Adenoviral Transduction of Mesenchymal Stem Cells: In Vitro Responses and In Vivo Immune Responses after Cell Transplantation
Source: PLoS One. 2012 Aug 6;7(8):e42662. doi: 10.1371/journal.pone.0042662 (PMC3412834; doi:10.1371/journal.pone.0042662)

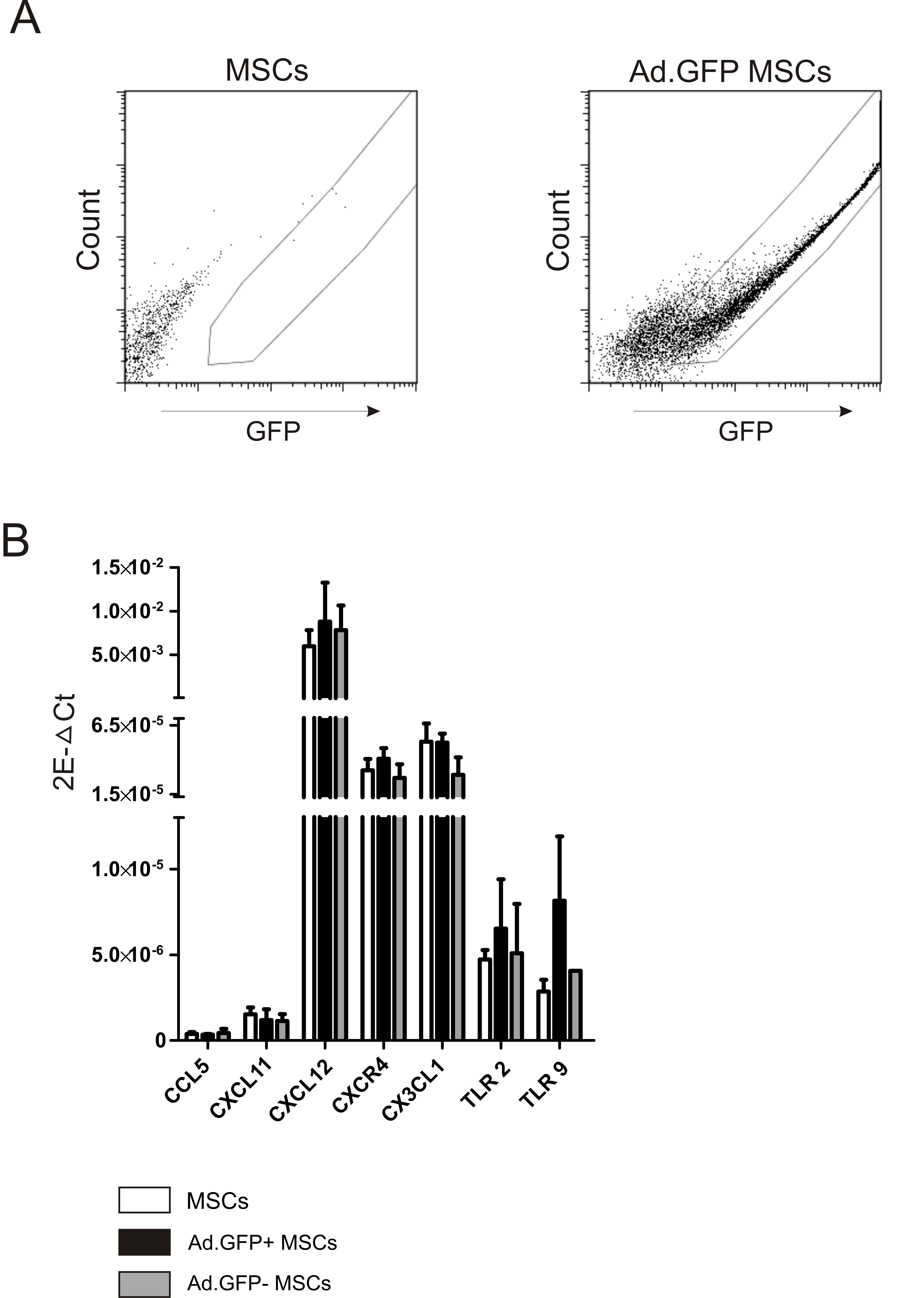

Supplement: Figure S1 — mRNA expression levels of TLRs, chemokines and chemokine receptors post-FACS sorting into Ad.GFP+ and Ad.GFP− fractions. (A) Representative dot plot and gating strategy of untransduced and Ad.GFP transduced MSCs as measured by flow cytometry. (B) Bar charts showing the mRNA expression levels of a representative selection of TLRs, chemokines and chemokine receptors post-FACS sorting into Ad.GFP+ and Ad.GFP− fractions, as measured by real-time RT-PCR. Shown is one representative experiment from two performed. 2E-ΔCT = 2−ΔCT→ number of copies of gene of interest relative to the number of copies of the internal control gene, β-actin. (TIF) [file pone.0042662.s001.tif]

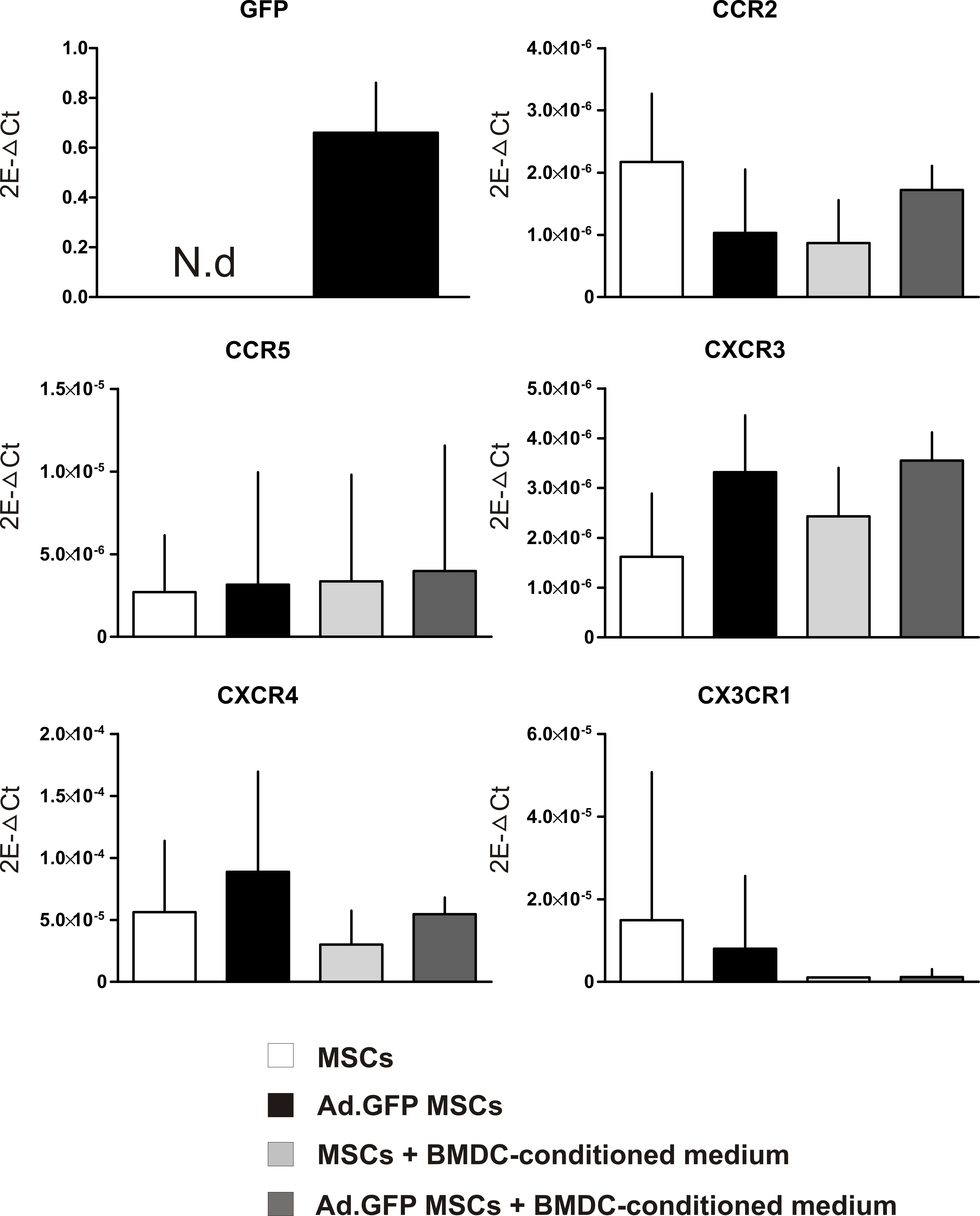

Supplement: Figure S2 — Chemokine receptor mRNA expression profile of untransduced and Ad-transduced MSCs following stimulation with BMDC-conditioned medium. RT-PCR analysis showing mRNA expression levels of a panel of chemokine receptors from untransduced and Ad-transduced MSCs in the presence or absence of BMDC-conditioned medium. Data shown are means ±SD of two separate isolations. 2E-ΔCT = 2−ΔCT→ number of copies of gene of interest relative to the number of copies of the internal control gene, β-actin. (TIF) [file pone.0042662.s002.tif]

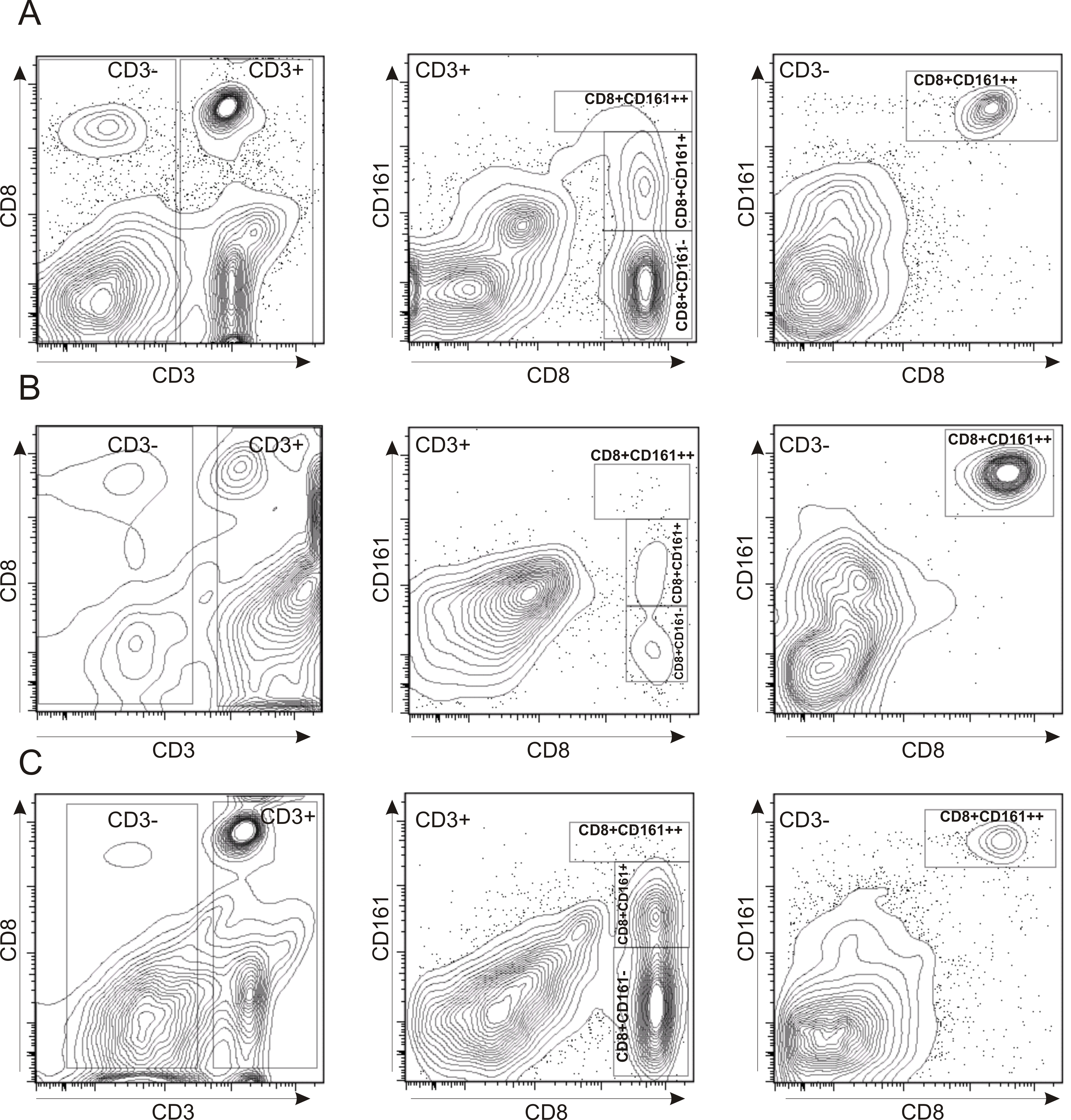

Supplement: Figure S3 — Gating strategy used for the analysis of cell distribution in PBMCs, lungs and spleens. Representative dot plots and gating strategy of (A) PBMCs, (B) lungs and (C) spleens from animals that each received an intravenous injection of 2×10^6^ Ad.GFP transduced MSCs (n = 4) or 2×10^6^ untransduced MSCs (n = 3). (TIF) [file pone.0042662.s003.tif]
